# Austrian farmers perception of new weeds

**DOI:** 10.1002/pei3.10129

**Published:** 2023-12-18

**Authors:** Michael Glaser, Franz Essl, Swen Follak

**Affiliations:** ^1^ Division of BioInvasions, Global Change & Macroecology, Department of Botany and Biodiversity Research University of Vienna Vienna Austria; ^2^ Vienna Doctoral School of Ecology and Evolution University of Vienna Vienna Austria; ^3^ Institute for Sustainable Plant Production, Austrian Agency for Health and Food Safety Vienna Austria

**Keywords:** agriculture, agroecosystems, climate change, invasive alien species, land use, management, online survey

## Abstract

The composition of weed floras in Central European fields has shifted creating a novel management issue: new weeds, that is, species that are currently spreading and increasing in impact. In their role as primary decision makers on the ground, farmers' perception of these new weeds plays a pivotal role in collecting information on their occurrence and control. We conducted an online survey to determine if Austrian farmers recognized 15 selected new weed taxa (12 species and 3 genera) from their farm. The 181 surveyed farmers also estimated the required management effort for these species and elicited their current management practices. Additional questions were posed to understand farmers' general perception of changes in the weed flora. We used a generalized linear mixed model to estimate differences in management effort and identify new weeds that merit monitoring and management programs. Two weed genera (*Fallopia* spp. and *Panicum* spp.) showed significantly higher than average management effort. The most commonly used management measures were manual removal, herbicide use and crop rotation. A majority of farmers reported changes in the weed flora; over two thirds reported new species and over one third reported new weeds that were difficult to control. In summary, our results suggest that respondents were aware of the challenges posed by new weeds but required more information on management and prevention strategies.

## Graphical abstract

We conducted an online survey among farmers in Austria in which we asked them if 15 pre‐selected emerging weed species occurred on their land as well as questions related to their management. Our results show that two genera (*Fallopia* spp. and *Panicum* spp.) are currently a major concern for Austrian farmers and that integrated, indirect weed management methods are under‐utilized.

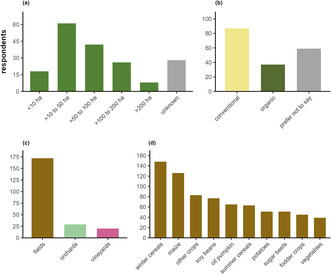

## 1 INTRODUCTION

Weeds cause substantial yield losses in agriculture as they compete with crop species (Oerke, 2006). In recent decades, certain weeds have become more frequent, including species that are difficult to control (Weber & Gut, 2005). These “new weeds” can be defined as weeds that are currently spreading and increasing in impact. Changes in the importance of certain weed species have been linked to changes in agricultural practices (Moss, 2017; Storkey et al., 2010). For example, intensive maize cultivation and the use of certain herbicides have benefitted panicoid grasses, such as *Echinochloa crus‐galli* (L. P. Beauv.) (von Redwitz & Gerowitt, 2017). Some weeds could benefit from climate change (Ramesh et al., 2017) which may enable species not seen as harmful in the past to become harmful weeds (Groves, 2006) and expand into regions that were previously climatically unfavorable. Additionally, climate change may affect weed‐crop competition and herbicide efficacy (Zimdahl, 2007b; Ziska, 2016). The number of alien species is rapidly increasing in Central European fields (Pyšek et al., 2005) and as the impacts of alien species are associated with considerable lag times (Rouget et al., 2016), species already present could be future weeds.

Farmers need to be aware of these species because prevention is the most effective management approach (Finnoff et al., 2007). Therefore, new weeds should be identified early and appropriate control strategies should be developed and made available to farmers.

Farmers are an important source of information on problematic weeds and their control due to their intimate knowledge of weed occurrences in their fields (Loux & Berry, 1991). Eliciting their perspective on weeds using surveys or in‐depth interviews can help to gain detailed information on, for example, reasons for species' introduction and spread, overall preferences for weed management as well as specific tactics to control certain weed species (e.g., Gibson et al., 2005; Wossink et al., 1997).

The study is aimed at understanding farmers' perspectives on new weeds – their occurrence and management effort – as well as providing a possible basis for integrating these perspectives into detection and management activities. More specifically, we address the following questions on the basis of an online survey shared among farmers in Austria:

- Which new weeds are most commonly recognized?
- How do farmers estimate the effort required to manage new weeds and which factors cause differences in this estimation?
- What are farmers' perceptions of changes in the weed flora and what potential reasons for this do they report?
- What direct and indirect weed management methods are currently applied?

## 2 MATERIALS AND METHODS

### 2.1 Study area

**FIGURE 1:**
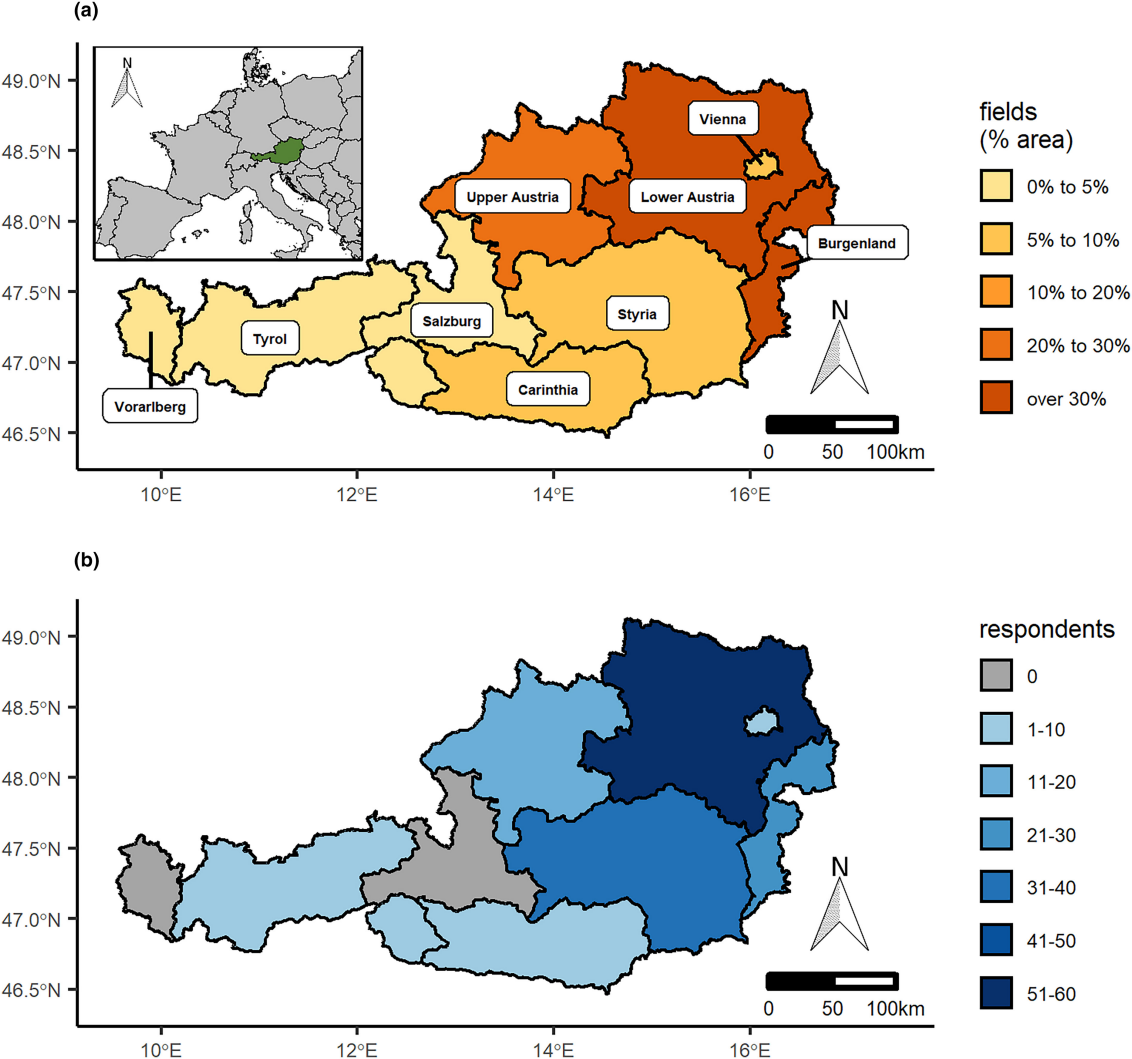
(a) Area used as fields as percentage of total area of the nine Federal Provinces of Austria. Insert plot shows the location of Austria in Europe. (b) Number of respondents (blue color scale) or lack thereof (gray).

This study was conducted among farmers in Austria (Figure 1a), a medium‐sized (c. 84,000 km^2^) landlocked Central European country. The prevailing climate is temperate with warm summers and cool winters in the lowlands, while the Austrian Alps and the Bohemian massif exhibit a cooler climate. An area of 1,344,000 ha (16%) of the Austrian territory is used as fields predominantly for the production of cereal, fodder, and oil crops. Less than one percent of Austria is covered by vineyards and orchards. The total area under organic cultivation was ca. 287,000 ha in 2020 (BLMRT, 2021). The diverse climate and agricultural land use alongside widespread organic farming create a broad array of different ecological niches. Together with its position along trade routes, an important corridor for the introduction and spread of weeds, this makes Austria an excellent case‐study for new weeds.

### 2.2 Selection of new weeds

We pre‐selected a list of weed species to include in the survey because farmers often use local common names so identification among similar species can be difficult. Thus, we considered a pre‐selection of species based on expert knowledge and stakeholder input a reasonable trade‐off compared to farmers listing possibly unidentifiable common names in open questions. To include relevant stakeholders in our selection early on, plant protection experts from all Austrian Federal Provinces (Figure 1a) received a form to submit candidate species that have, based on their experience, become more abundant and/or difficult to control. In a second step, we identified species from the List of Invasive Alien Plants and the Alert list of the European and Mediterranean Plant Protection Organization (EPPO, 2022) relevant to Austrian agriculture (e.g., *Helianthus tuberosus*). We limited our list to 15 species because a questionnaire containing too many species may have led to respondents abandoning the questionnaire prematurely.

As some weed genera include species which require expert knowledge for identification, we grouped these species into their genera for the purpose of the survey, that is, *Amaranthus* spp., *Fallopia* spp. and *Panicum* spp. Because of the prevalence of potential new weeds in the genus *Amaranthus* (Milani et al., 2020), we included this genus in our pre‐selection, even though some species are already well established as weeds. In contrast, species grouped into *Fallopia* spp. and *Panicum* spp. are in a far earlier stage of spread in Austrian fields (Hügin, 2010; Kadlecová et al., 2022). While the grouping of species into genera comes with some taxonomic uncertainty about the recognized species, the images we provided for identification were from those species relevant as weeds. To emphasize this to respondents, a statement was included, that only visual recognition of the species was relevant. The selected new weeds span several families and a range of growth and life forms (Table 1), most of them are alien, thermophilic, and nitrophilic (Tichý et al., 2023).

**TABLE 1:** New weeds (12 species and three genera) selected for the online survey (selection criteria marked with *), their key traits and reasons for inclusion. growth: a—annual, b—biennial, p—perennial, life form (Raunkiær, 1907): g—geophyte, h—hemicryptophytes, t—therophytes; t, N: Ecological Indicator Values (Tichý et al., 2023) for temperature and nutrients, respectively; residence time: arc—archaeophyte, neo—neophyte, mix—species group of mixed origin (sensu Pyšek et al., 2004), intro: earliest first record for Austria PPE—species mentioned by regional plant protection experts, EPPO—species listed on the EPPO List of Invasive Alien Plants and Alert list (EPPO, 2022).

Family	Taxon	Growth	Life form	T	N	Residence time	Intro	PPE*	EPPO*
Amaranthaceae	*Amaranthus* spp.	a, p	t, h	8	7.8	neo		y	y^†^
Apocynaceae	*Asclepias syriaca* L.	p	g	6.9	5	neo	1800	y	y
Asteraceae	*Ambrosia artemisiifolia* L.	a	t	7.2	6.2	neo	1883	y	y
Asteraceae	*Helianthus tuberosus* L.	p	g	6.9	7.7	neo	1700		y
Asteraceae	*Lactuca serriola* L.	a	t	x	x	arc		y	
Asteraceae	*Xanthium orientale* agg.	a	t	x	6.5	neo	n.a.	y	
Brassicaceae	*Descurainia sophia* (L.) Webb ex Pranti	a	t	x	6.3	arc			y
Cyperaceae	*Cyperus esculentus* L.	p	g	9.5	6.0	neo	1900	y	y
Malvaceae	*Abutilon theophrasti* Medik.	a	t	x	x	neo	1873^‡^	y	
Phytolaccaceae	*Phytolacca americana* L.	p	h	x	6.0	neo	1800	y	
Poaceae	*Panicum* spp.	a, p	h	8	6	neo		y	
Poaceae	*Sorghum halepense* (L.) Pers.	p	g	7	7	neo	1899	y	
Polygonaceae	*Fallopia* spp.	p	h	x	6.1	mix		y	y
Solanaceae	*Datura stramonium* L.	a	t	x	7.9	neo	1583	y	
Solanaceae	*Solanum carolinense* L.	p	h			neo	1998^§^		y

^†^ Only some species in genus listed.

^‡^ Follak et al. (2014).

^§^ Zernig et al. (2015).

### 2.3 Survey design & distribution

Farmers were presented with at least two images of each selected new weed (Table 1) and asked whether this species was present on their farms. Subsequently, farmers were asked to estimate the management effort required per every recognized species on a five‐part‐scale from low (i.e., no additional management effort) to high (i.e., requires large additional management effort). Additional questions were aimed at (i) characterizing farms operated by respondents (e.g., farm size, conventional vs. organic, land use type, major crop types), (ii) direct and indirect methods used for weed control by choosing from a list of 12 common methods (Zwerger & Ammon, 2002) and (iii) farmers' perception of the changes in weed flora and the drivers they identified. Farmers were given the option to suggest additional new weeds and give additional input on control methods as well as the option to “prefer not to say” when answering potentially sensitive questions. For the complete questionnaire see Table S1. The survey was distributed via key stakeholder groups (e.g., Provincial Chambers of Agriculture) from January 18, 2022 to April 30, 2022 resulting in almost 30,000 stakeholders receiving an invitation to participate in the survey. For a full list of stakeholders and reach estimates see Table S2. Survey design, hosting, and data collection were done via SoSciSurvey (www.soscisurvey.de) following current data protection guidelines.

### 2.4 Statistical analysis

As we also wanted to know if organic farms were more affected by new weeds than conventional farms (Gallandt, 2014), we compared the total number of new weeds recognized with a Kruskall–Wallis test. To analyze the differences in management effort between organic and conventional farms, a binomial family generalized linear mixed model (Bolker et al., 2009) was fitted with management effort as a response, farm type, and species as fixed effects as well as individual farmers as a random effect to correct for their varying baselines when judging weed management effort. As no reference group can be defined, sum contrasts (Schad et al., 2020) were used for species effects and significance thus should be interpreted as deviances from the mean across all species (i.e., a significant positive effect indicates a species is harder to manage than average). The farm type (i.e., organic vs. conventional) showed no significant influence as a predictor, so it was removed and we report here differences in estimated species' management effort. All analyses presented here were performed in R (R Core Team, 2021).

## 3 RESULTS

Of the 216 responses, 35 were only partly answered and thus excluded from analyses; the final data set thus covers 181 responses. These come from seven of the nine Austrian Federal Provinces, mostly from Lower Austria (60), followed by Styria (40), the least from Tyrol and Vienna (two each). A majority of respondents were from the eastern part of Austria (Figure 1b), reflecting the spatial distribution of fields in Austria (Figure 1a).

**FIGURE 2:**
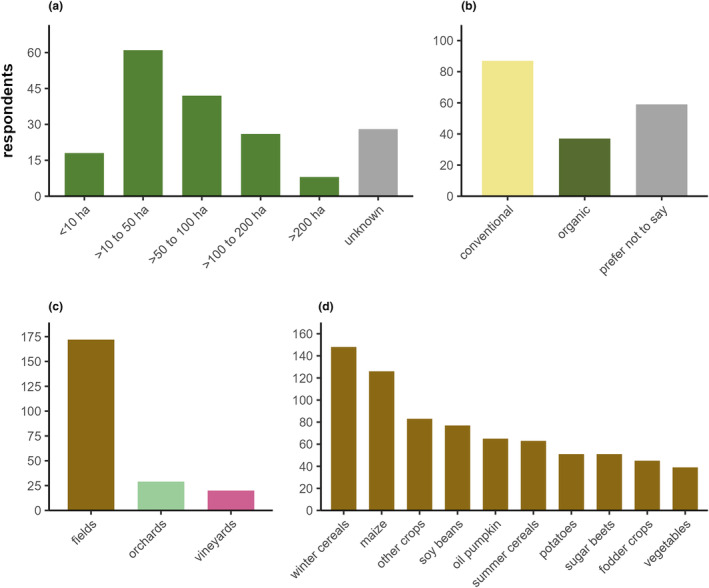
Summary of respondent data (*n* = 181) by (a) farm size (total: ca. 11,000 ha), (b) farming practice, (c) major land use type, and (d) major crop types.

Most respondents (33%) managed farms with a size between 10 and 50 ha (median size: 50 ha) and used conventional farming practices (43%), cultivated fields (94%) and there, mostly grew winter cereals (83%) and maize (70%) (Figure 2a–d, respectively). An overview of the data used for analysis is given in Table S3.

### 3.1 Recognized new weeds

**FIGURE 3:**
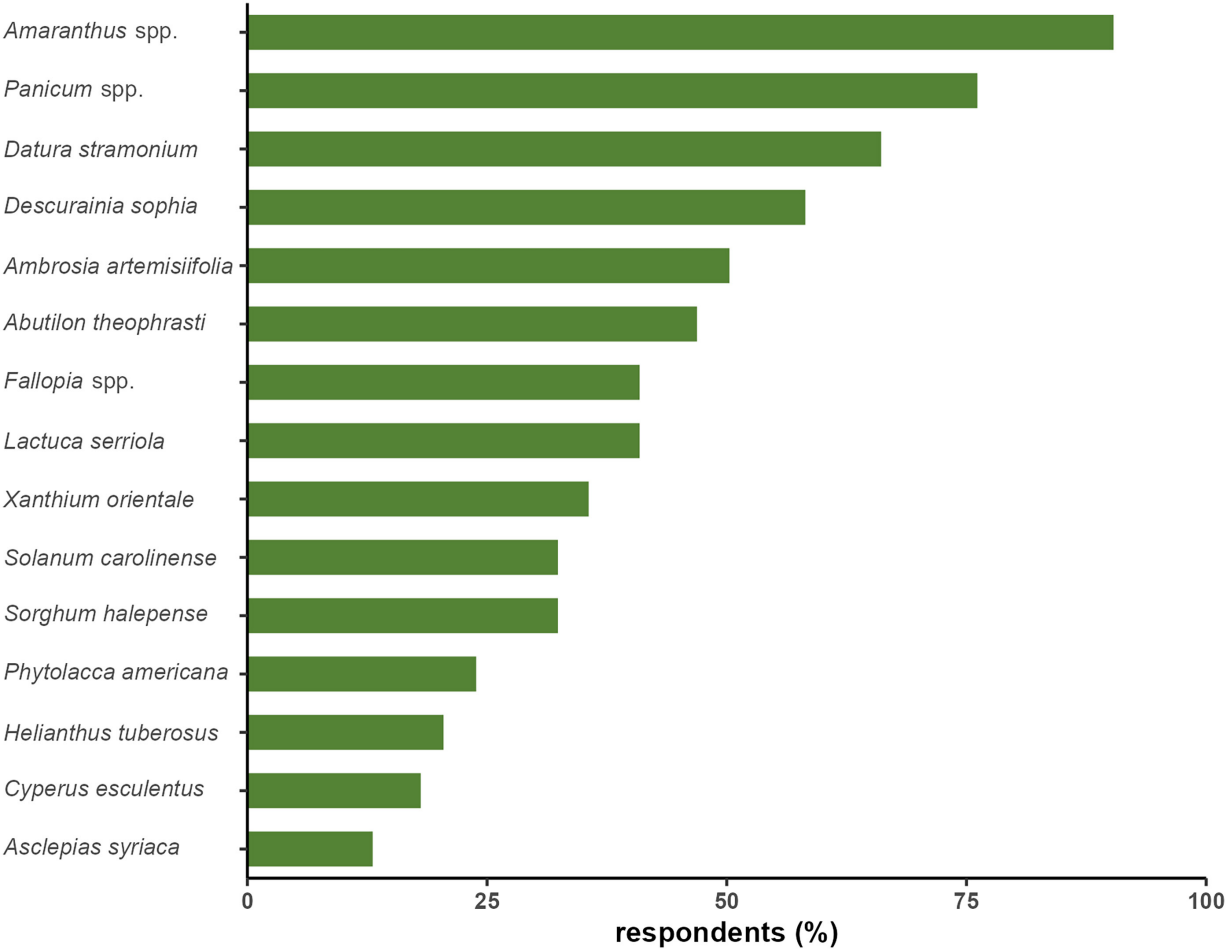
Percentage of respondents *n* = 181) that recognized the selected new weeds on their farm. For more details on the species see Table 1 and for the image used for aiding identification in the survey, see Table S1.

A median of six species was recognized by respondents – less than 5% (7) recognized none. The most commonly observed species were *Amaranthus* spp. Followed by *Panicum* spp. And *Datura stramonium* which were recognized by 88% (161), 74% (135), and 65% (119) of respondents, respectively. The species least commonly recognized were *Cyperus esculentus* and *Asclepias syriaca*, which were recognized by 18% (33) and 13% (24) of respondents, respectively (Figure 3). No significant differences could be found between the number of species recognized between organic and conventional farms (Kruskal–Wallis test, *p* = .370).

In the open questions, 15% of respondents (27) named a total of 35 additional species. While these were mainly common weeds (e.g., *Chenopodium album*), mentions did include some species (e.g., *Impatiens glandulifera*) that should be observed further because they are gaining increased importance as weeds (EPPO, 2022).

### 3.2 Estimated management effort

**FIGURE 4:**
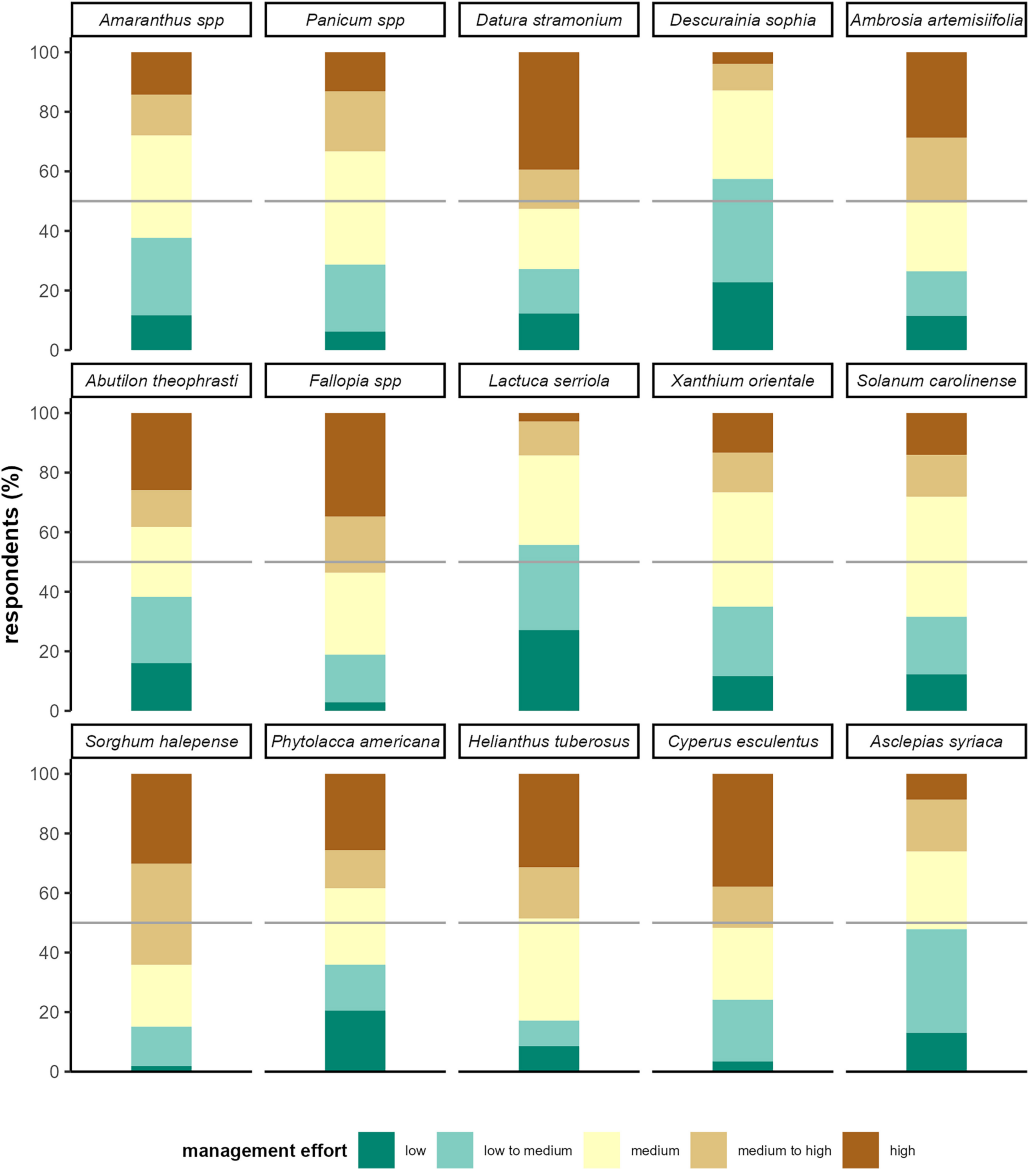
Reported management effort to control the selected new weeds (*n* = 181), gray line indicates 50%.

A majority of respondents reported medium‐ to high‐management effort for *Sorghum halepense* (64%), *Datura stramonium* (54%), *Cyperus esculentus* (53%), *Fallopia* spp. (53%), and *Ambrosia artemisiifolia* (51%). According to the respondents, *Descurainia sophia* (57%), *Lactuca serriola* (57%), *Asclepias syriaca* (45%), and *Amaranthus* spp. (38%) are rather easy to control with most respondents reporting medium‐ to low‐management effort (Figure 4).

The model revealed significantly higher estimated management effort for *Fallopia* spp. and *Panicum* spp. as well as lower estimated management effort for *Lactuca serriola* and *Descurainia sophia* (Table 2). Thus, the former two species can be considered significantly harder than average, the latter two as significantly easier than the average to manage and the other species were not significantly different from average in their estimated management effort.

**TABLE 2:** Summary of the mixed model including new weeds as fixed and respondents as random effects to correct for individual differences in estimated management effort.

Predictors	Estimate	Standard error	Z‐value	*p*‐value
Intercept	3.172	0.307	10.337	<.001***
*Abutilon theophrasti*	−0.640	0.391	−1.637	.102
*Amaranthus* spp.	0.085	0.325	0.262	.793
*Ambrosia artemisiifolia*	0.022	0.409	0.053	.958
*Asclepias syriaca*	−0.489	0.754	−0.649	.516
*Cyperus esculentus*	0.951	1.070	0.889	.374
*Datura stramonium*	−0.090	0.365	−0.246	.806
*Descurainia sophia*	−1.299	0.337	−3.849	.001***
*Fallopia* spp.	1.815	0.752	2.413	.016**
*Helianthus tuberosus*	0.196	0.716	0.273	.785
*Lactuca serriola*	−1.524	0.380	−4.007	<.001***
*Panicum* spp.	0.933	0.428	2.180	.029**
*Phytolacca americana*	−0.801	0.514	−1.557	.120
*Solanum carolinense*	−0.414	0.488	−0.848	.397
*Sorghum halepense*	1.669	1.030	1.620	.105
*Xanthium orientale*	−0.414	5.090	0.081	.935

*Note*: Significant effects (i.e., significant deviations from the mean over all species) in boxes, significances are given as: **p < .05, ***p < .01 and significant species are shown in bold.

### 3.3 Farmers perceptions of changes in the weed flora and reasons for it

**FIGURE 5:**
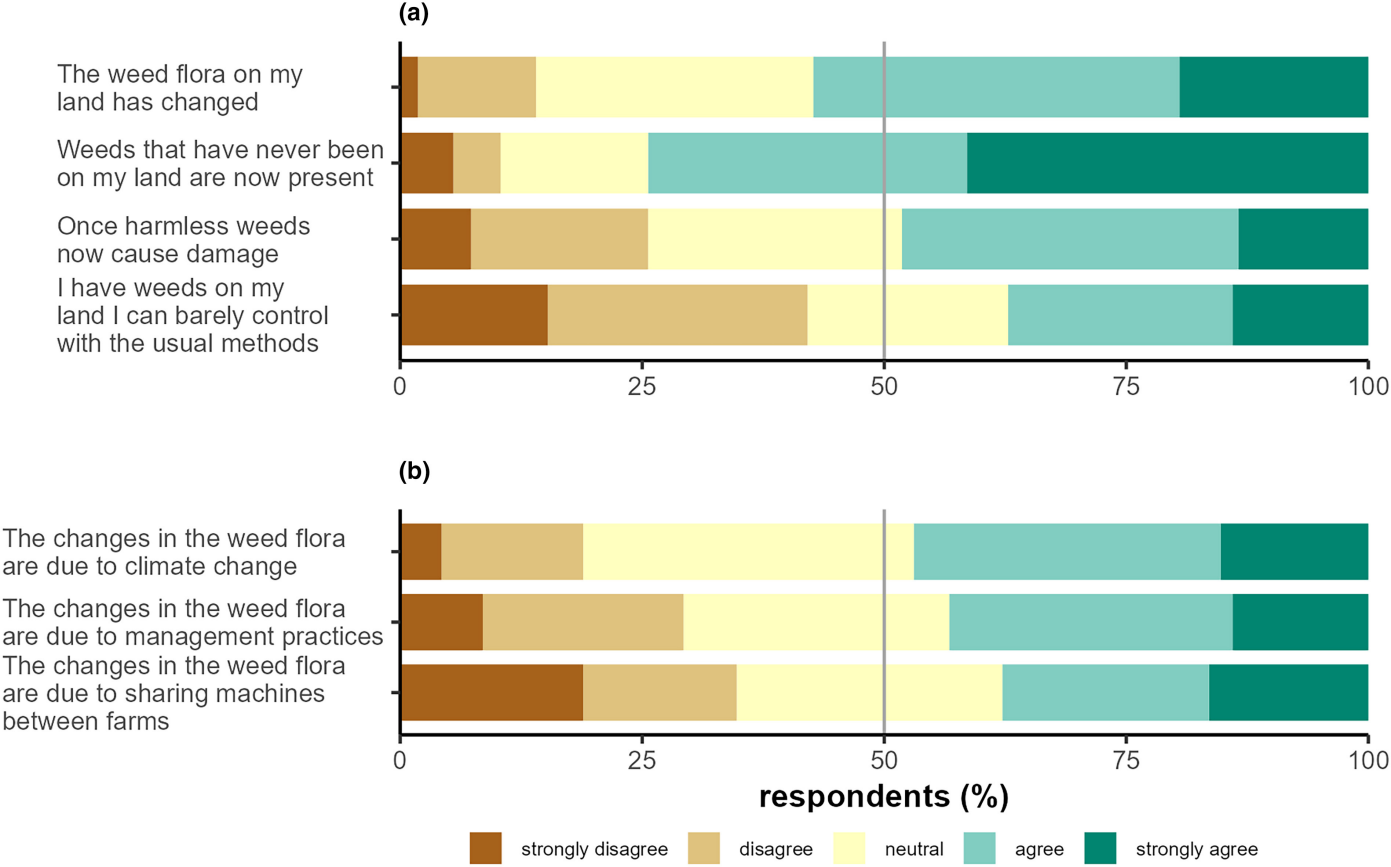
Farmers (*n* = 164) perceptions of changes in the weed flora: (a) Farmers agreement with statements about change and (b) their agreement with potential reasons for these changes. Gray line indicates 50%.

More than half of respondents (54%) reported the weed flora on their land having changed and more than two thirds (69%) also reported new weeds appearing (Figure 5a). The changes in the weed flora are also underscored by almost half of respondents (44%) reporting that weeds now causing damage used to be seen as harmless and over one third of respondents (35%) reporting weeds that are difficult to control (Figure 5a).

Respondents rated the plausibility of possible causes for changes in the weed flora. Slightly less than half (43%) of the farmers report that climate change is causing alterations in the weed flora. Less than half of the farmers (40%) agreed that changes in the weed flora can be attributed to the management practices used. Only one third believed machine sharing between farms (Figure 5b) was a cause for the spread of new weeds.

### 3.4 Weed management methods

**FIGURE 6:**
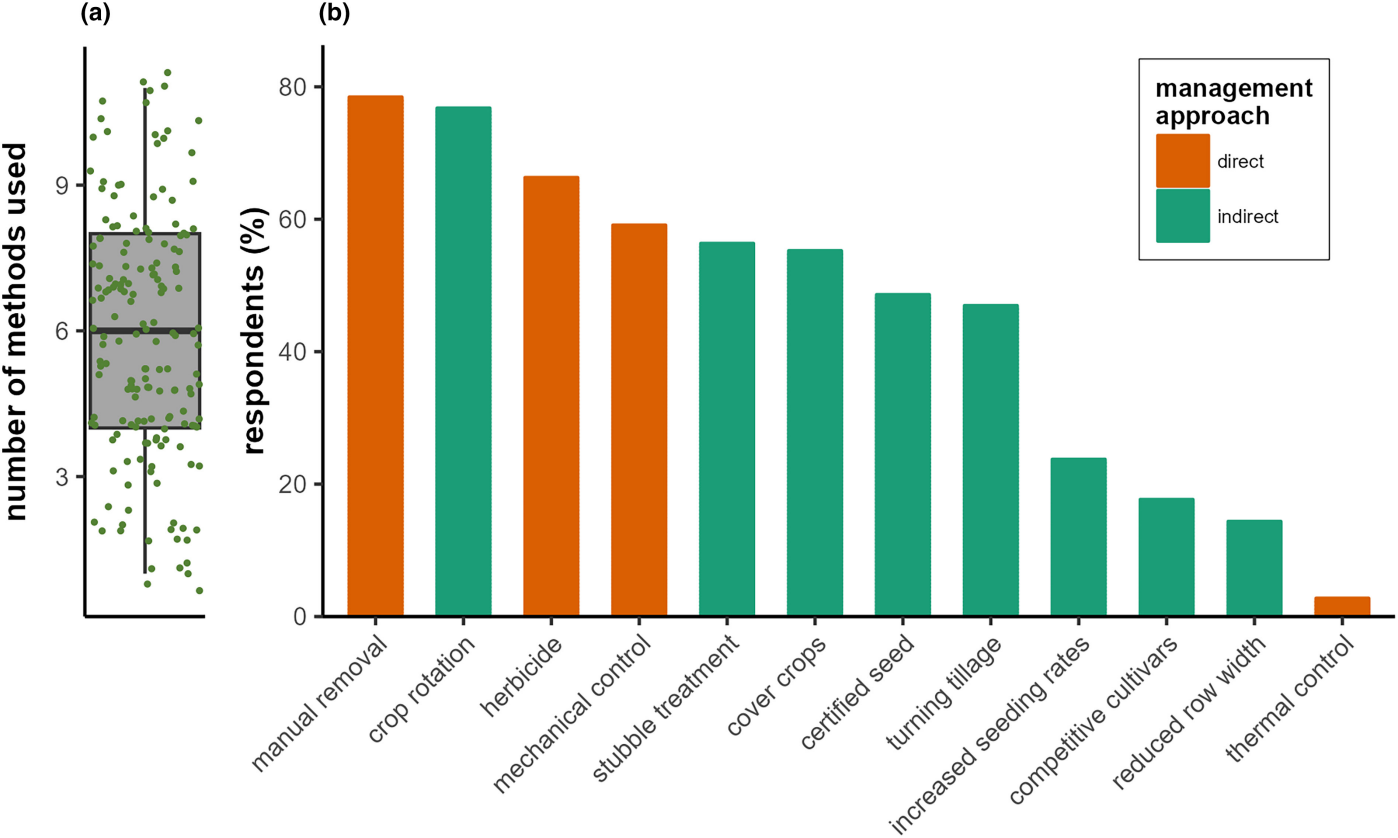
Overview of weed management methods used by the respondents (*n* = 181): (a) number of management methods used (points) as well as their distribution (box plot) and (b) percentage of respondents reporting the use of the given management methods, color corresponds to classification of management methods in Zwerger and Ammon (2002).

The median number of given weed management methods used per farm was six (Figure 6a), with 19 respondents using two or fewer and 13 respondents using 10 or more of the suggested methods. Only five additional open answers were given, suggesting the use of mulch for weed control. The vast majority of farmers (84%) used two of the three most commonly used weed management methods (Figure 6b): manual removal, crop rotation and herbicides. Less frequently used methods were mechanical control, stubble treatment (i.e., soil cultivation after harvest), cover crops, certified seed, and turning tillage. Even less frequently used were changes in crop production methods, such as increased seeding rates, cultivation of competitive cultivars, and reduced row width. Thermal weed control was by far the most rarely used weed control method.

## 4 DISCUSSION

This study aimed to assess Austrian farmers' perception of the potential threat by new weeds and to integrate practitioners' experiences with weed science. It addressed questions regarding changes in the weed flora, the recognition, and management effort for selected species as well as current management practices. More than half (58%) of respondents reported changes in the weed flora on their land and close to half reported new species occurring as well as weeds once deemed harmless now causing damage (both 48%). Regarding the weed management methods practiced, we found that most farmers rely on a small set of methods, suggesting the limited adoption of integrated weed management (Chauhan et al., 2017; Swanton et al., 2008).

### 4.1 Perceptions on changes in the weed flora

Farmers seem to some degree aware of changes in the weed flora and the appearance of new weeds causing significant damage. This awareness is probably not yet sufficient and farmers should receive in‐depth information on this issue. In this respect, the data suggest that farmers are not fully aware of the underlying causes of the changes in the weed flora. Less than 50% of respondents agreed that changes can be attributed to management practices or machine sharing between farms even though both are important dispersal vectors for weed propagules (Benvenuti, 2007; von Redwitz & Gerowitt, 2017). Likewise, Wilson et al. (2008) concluded from their survey that farmers largely attribute the introduction and spread of new weeds to factors outside their control (e.g., natural spread). Less than half of the respondents identified the role of climate change, although the introduction and the spread of new weeds are favored by climate change in Central Europe (Peters et al., 2014).

### 4.2 Problematic new weeds

*Fallopia* spp. and *Panicum* spp. showed significantly higher than average management effort (Table 2) and are both considered competitive with high‐reproduction rates (Kadlecová et al., 2022; Zimdahl, 2007a). Additionally, respondents reported high management effort for two species with human health impacts: *Datura stramonium* (41% of 119 farmers who had occurrences of the species) and *Ambrosia artemisiifolia* (51% of 89 farmers) which both often require specific and time‐consuming measures to mitigate their negative impacts. Other problematic species were *Cyperus esculentus* (40% of 33 farmers with occurrences claiming high‐management effort), *Helianthus tuberosus* (31% of 36 occurrences) and *Sorghum halepense* (30% of 57 occurrences) – all perennials, challenging to manage due to extensive vegetative reproduction and capacity for regeneration. *Cyperus esculentus* and *Sorghum halepense* were recognized by fewer farmers, though other studies showed a rapid recent increase in fields in Austria (Follak et al., 2017). Our results also indicate that *Fallopia* spp. and *Helianthus tuberosus* increasingly infest fields, which can be corroborated by observations from neighboring countries (e.g., Czech Republic: Kadlecová et al., 2022). This is most likely due to high‐propagule pressure from large populations in riparian and anthropogenic habitats close to fields (Follak pers. obs., 2022). It is noteworthy that several highly thermophilic species (e.g., *Sorghum halepense*, *Ambrosia artemisiifolia*) already have a reportedly high‐management effort. The species will most likely continue to spread in Austria due to climate change (Kleinbauer et al., 2010).

### 4.3 Consequences for weed management

Farmers unaware of new weeds may attempt to use inadequate methods, wasting their efforts and possibly enabling spread to other farms. This is underscored by the fact that over one third of respondents reported having problems with weed control. Most farmers use between four and six weed management methods; however, their efficacy depends on the species, its level of infestation as well as the appropriate timing and application of management methods. The most commonly mentioned method was manual removal, even though this method is not feasible for larger weed infestations. It may indicate that populations of the studied species are often still rather small in fields. Herbicide use and mechanical control were two additional methods regularly used. Some species (e.g., *Cyperus esculentus*) are generally difficult to control by these methods, while for others (e.g., *Phytolacca americana*), there is a lack of data on herbicide efficacy, especially under the conditions in Austria. Among the least‐used weed control methods were those that enhance the competitive ability of the crop (i.e., row width, seeding rates, competitive cultivars). It is well known that these methods can contribute to effective weed regulation (Drews et al., 2009). It was to be expected that thermal methods are rarely used. While their effectiveness depends on specific factors (e.g., developmental stage, weather conditions) and their application is demanding, they can provide an effective method for individual patches or comparably small scales (Bauer et al., 2020).

Taken together, our results regarding weed management methods show that farmers rely largely on direct control methods with only one indirect method, crop rotation, being regularly used. Other surveys also show that farmers focus more on control than prevention (Wilson et al., 2008). This emphasizes the need for the evaluation of integrated weed management approaches for the control of new weeds.

### 4.4 Limitations

Given the wide distribution of the survey link, the sample size (*n* = 181) was rather small. Sample size could have reduced the significance of the results, for example, in estimated management effort for species with low reported recognition (e.g., *Asclepias syriaca*, *n* = 23). This may also explain that no difference in management effort or number of new weeds was found between organic and conventional farms. However, the majority of respondents was from those federal provinces with the largest proportion of fields. Therefore, we believe that the results still provide valuable insights into Austrian farmers' perceptions of new weeds.

The pre‐selection of species may have led to relevant species having been excluded, however, we believe that all important new weed species have been taken into account as the list (Table 1) combined information from plant protection experts as well as stakeholders. It contains species that are at the beginning of their spread (e.g., *Asclepias syriaca*) to species that already have an impact on crop production (e.g., *Abutilon theophrasti)*. Additionally, respondents did not mention many other new weeds frequently in the open question section confirming our pre‐selection was relevant.

## 5 CONCLUSIONS

The spread of the new weeds identified is likely to continue in agricultural areas of Austria due to climate and land use change. The most problematic new weeds according to the perception of survey respondents were *Fallopia* spp., *Panicum* spp., *Datura stramonium*, *Cyperus esculentus*, and *Sorghum halepense*. Farmers need to be made aware of these species, especially in those areas of Austria where they are currently rare or not yet present. Here, taking preventive measures are important options for them (e.g., regular field observations, cleaning machinery). Future studies could use species distribution modeling to identify areas at high risk of these new weeds occurring. Monitoring and the adoption of rapid response strategies can prevent establishment and/or limit their spread. Farmers who already have major problems with new weeds need concepts to control them, which should be tailored to the conditions in Austria. Such strategies are already available for some species (e.g., *Ambrosia artemisiifolia*, EPPO (2021); *Cyperus esculentus* Bohren, (2016) while strategies for other species (e.g., *Asclepias syriaca*) still need to be developed.

## FUNDING INFORMATION

This study was funded by the Austrian Climate Research Program [FA772033 “AgriWeedClim”]. MG and FE appreciate funding by the Austrian Science Foundation (FWF) [grant no. I 5825‐B].

## CONFLICT OF INTEREST STATEMENT

The authors declare no conflicts of interest.

## Supporting information


Table S1.
Table S2.

Table S3.

## Data Availability

The data used from the survey is provided in Table S3 in an anonymized form.
