# Supplementary material for: Austrian farmers perception of new weeds
Source: Plant Environ Interact. 2023 Dec 18;5(1):e10129. doi: 10.1002/pei3.10129 (PMC10840368; doi:10.1002/pei3.10129)
Supplement: Supplementary file 1 — Table S1. Table S2. [file PEI3-5-e10129-s001.docx]

Austrian Farmers Perception of New Weeds

Michael Glaser^*, 1, 2^ (orcid: 000-0002-4695-6150,

Franz Essl^1, x^ (orcid: 0000-0001-8253-2112)

and Swen Follak^3, x^ (orcid: 0000-0002-1788-0572)

^1^ BioInvasions, Global Change, Macroecology Group, Department of Botany and Biodiversity Research, University of Vienna, Austria

^2^ Vienna Doctoral School of Ecology and Evolution, University of Vienna, Austria

^3^ Institute for Sustainable Plant Production, Austrian Agency for Health and Food Safety, Vienna, Austria

^*^ corresponding author

^x^ these two authors are joint senior authors

Correspondence

Michael Glaser, Rennweg 14, 1030 Wien

Email: [michael.glaser@univie.ac.at](mailto:michael.glaser@univie.ac.at)

Table S 1: Full questionnaire overview including English translation of questions. Answer type shows type of response possible

| **Question 1**  filter question to remove non-target respondents (i.e. those selecting "none of the above") | **German (original)** | **English (translated)** |
| --- | --- | --- |
|  | Mein Betrieb bewirtschaftet folgende Flächen... | I cultivate the following types of land... |
| **Answer type** | multiple choice | |
| **Answer** | **German (original)** | **English (translated)** |
|  | - Äcker - Obstgärten - Weingärten - keine davon | - fields - orchards - vineyards - none of the above |
| **Additional Information** | "none of the above" skip questionnaire, are thanked for participation and then discarded from sample. | |
| **Question 2** | **German (original)** | **English (translated)** |
|  | Mein Betrieb wirtschaftet... | I use ... farming practices |
| **Answer type** | single choice | |
| **Answer** | **German (original)** | **English (translated)** |
|  | - biologisch - konventionell - keine Angabe | - organic - conventional - prefer not to say |
| **Question 3** | **German (original)** | **English (translated)** |
|  | Auf meinen Äckern baue ich an... | On my fields I cultivate... |
| **Answer type** | Multiple choice with open text entry option "other". | |
| **Answer** | **German (original)** | **English (translated)** |
|  | - Wintergetreide - Sommergetreide - Körnermais - Körnererbsen - Ackerbohnen - Kartoffeln - Zuckerrüben - Raps und Rübsen - Sonnenblumen - Sojabohnen - Öl-Kürbis - Silo- und Grünmais - Kleegras - Gemüse (inkl. Freiland, unter Glas) - Sonstige Kulturen | - winter cereal - summer cereal - grain maize - grain peas - beans - potatoes - sugar beets - canola - sunflowers - soy beans - oil pumpkins - silo maize - clovergrass - vegetables (incl. outdoor under glass) - other |
| **Info Text 1** | **German (original)** | **English (translated)** |
|  | In den folgenden Fragen geht es um neue Unkräuter, die in der Landwirtschaft zugenommen haben und auch um Arten, die in den letzten Jahren neu aufgetreten sind. Wir zeigen Ihnen Fotos dieser Unkräuter und würden gerne wissen, ob Sie diese Art auf Ihrem Betrieb schon einmal gesehen haben, ob Sie die Namen erkennen ist dabei eher unwichtig*. Unkräuter haben viele verschiedene Namen und wir zeigen immer nur die (unserer Meinung nach) bekanntesten!* | The following questions deal with new weeds, that are increasing in numbers or have become naturalized recently. We will show you pictures of a species and would like to know if it occurs on your farm*. If you recognize the name or not is unimportant, weeds haven many different names and we only list the (in hour opinion) most commonly used ones!* |

| **Question S1-S15**  Questioins aimed at the occurrence of selected emerging weeds (Table 2) on resondents' farms. | **German (original)** | **English (translated)** |
| --- | --- | --- |
|  | Kommt die abgebildete Art auf den von Ihnen bewirtschafteten Flächen vor? | Does the species in the pictures occur on your farm? |
| **Answer type** | binary (Yes/No) | |
| **Additional Information** | All questions serve as filter questions for questions M1-M15 regarding the management estimate for emerging weed species (i.e., only farmers that had these species on their land were allowed to estimate management effort). | |
| **Question S1** | *Ambrosia artemisiifolia* | |
| **Additional Information** | Pictures of species to aid recognition. | |
| 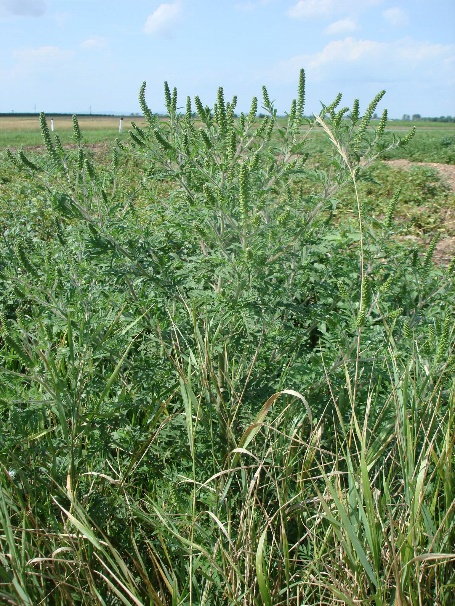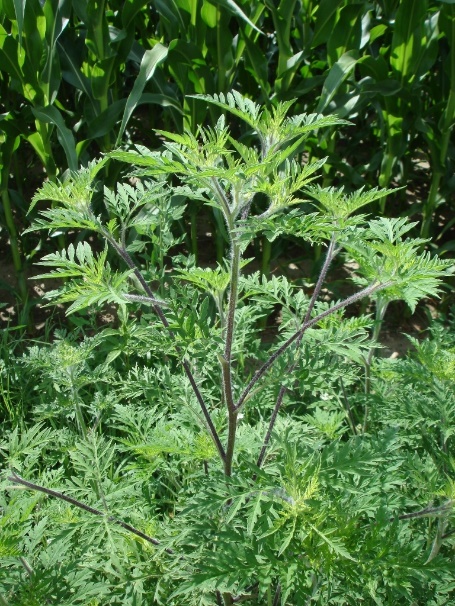 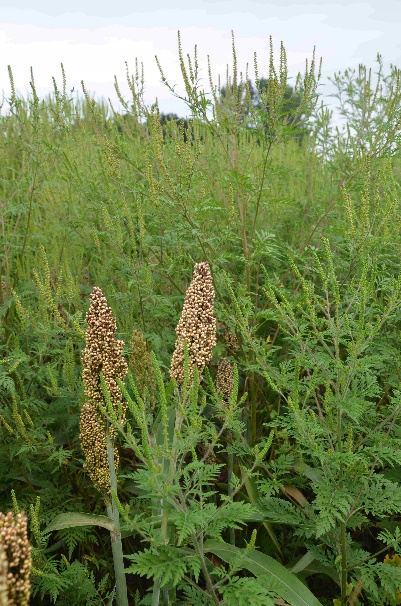  image credit: Swen Follak (all) | | |
| **Question S2** | *Cyperus esculentus* | |
| **Additional Information** | Pictures of species to aid recognition. | |
| 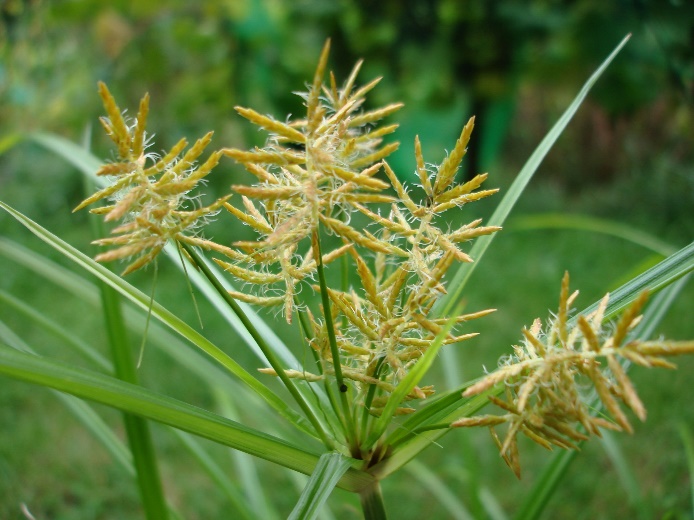 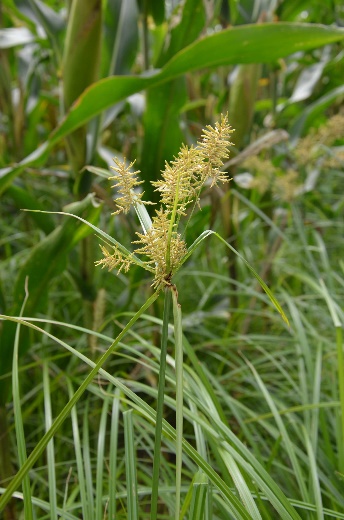 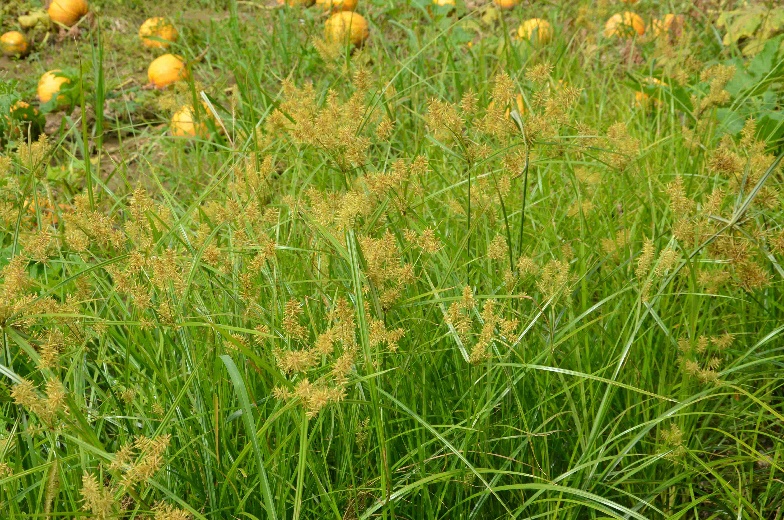  image credit: Swen Follak (all) | | |
| **Question S3** | *Xanthium strumarium* | |
| **Additional Information** | Pictures of species to aid recognition. | |
| 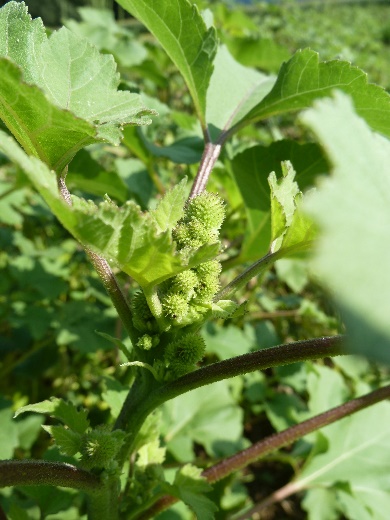 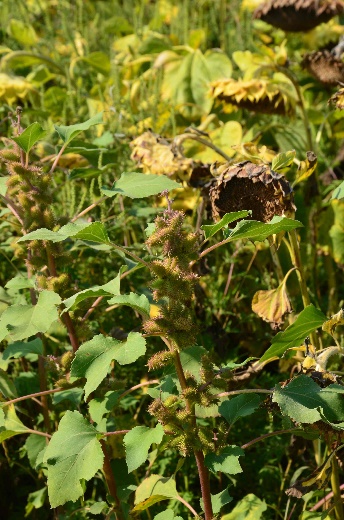 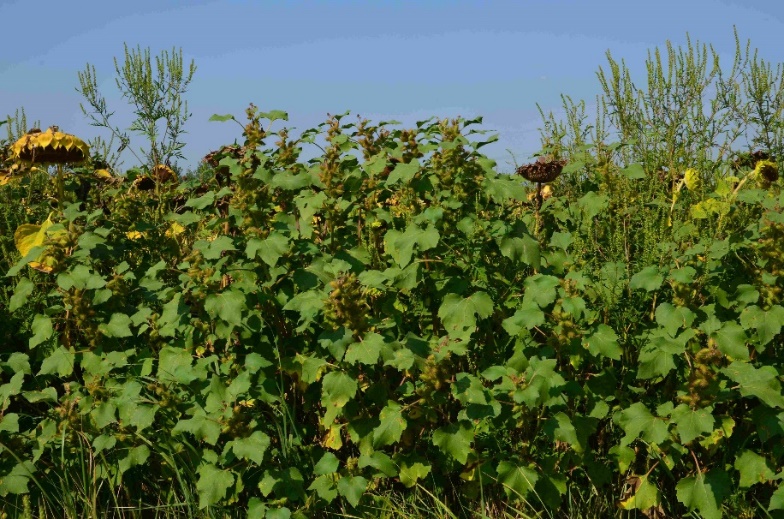  image credit: Swen Follak (all) | | |
| **Question S4** | *Datura stramonium* | |
| **Additional Information** | Pictures of species to aid recognition. | |
| 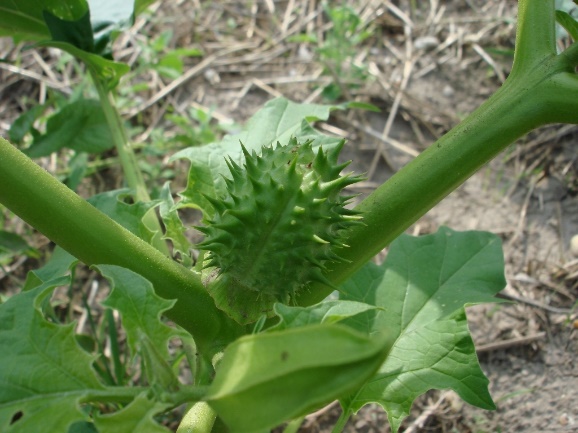 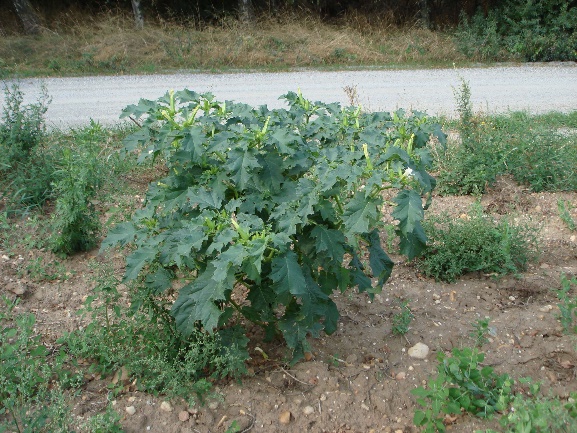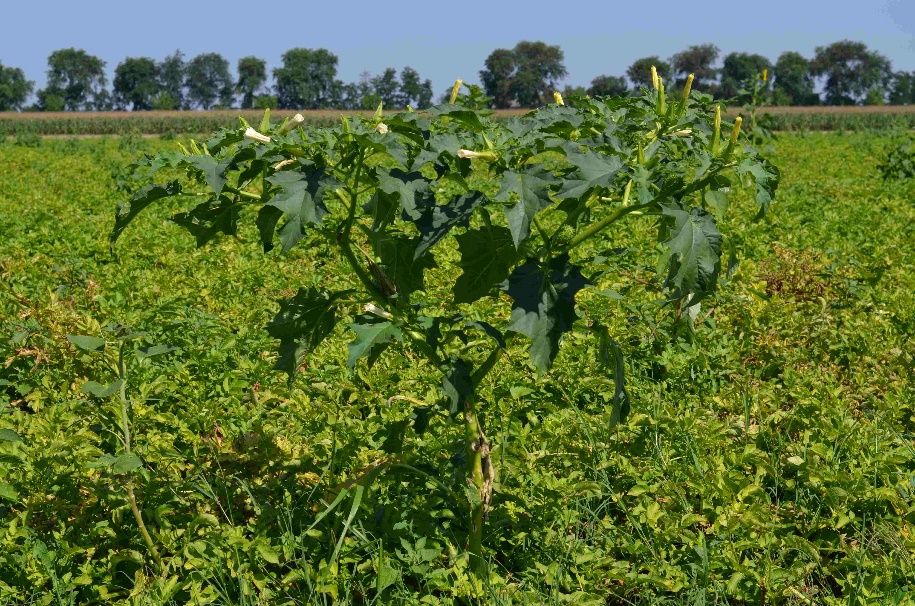  image credit: Swen Follak (all) | | |
| **Question S5** | *Phytolacca americana* | |
| **Additional Information** | Pictures of species to aid recognition. | |
| 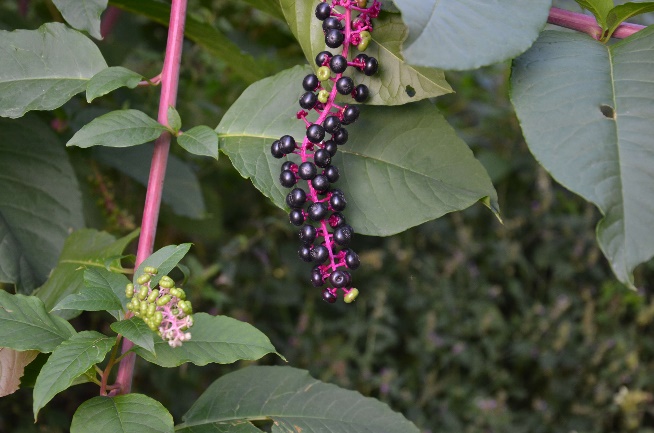 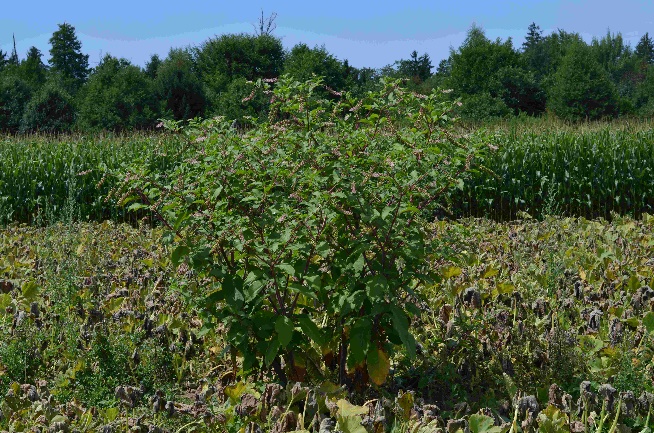  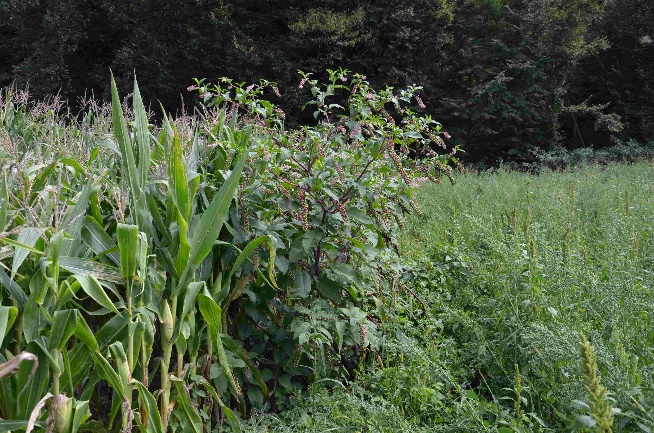  image credit: Swen Follak (all) | | |
| **Question S6** | *Sorghum halepense* | |
| **Additional Information** | Pictures of species to aid recognition. | |
| 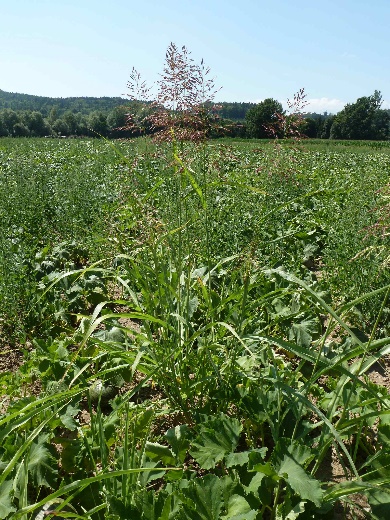 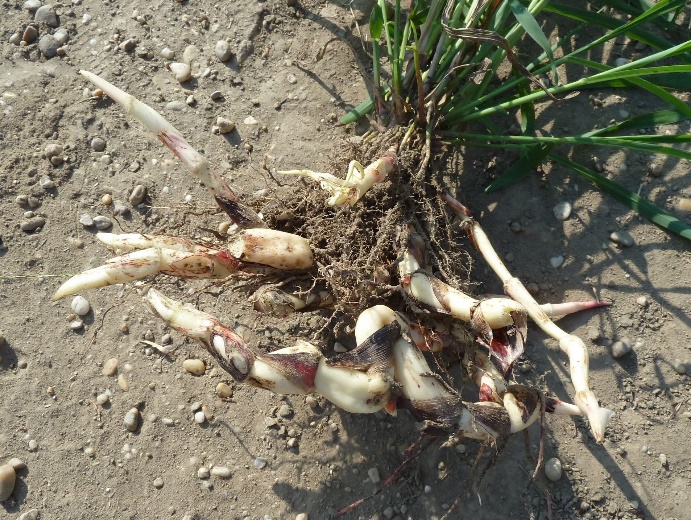  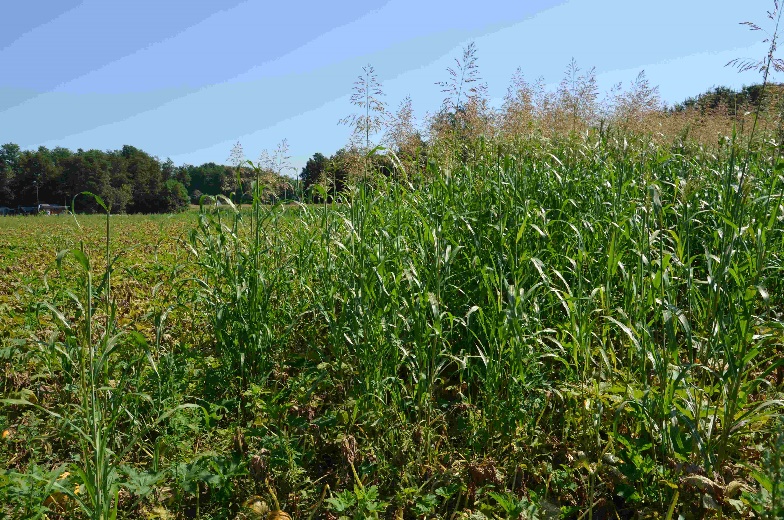  image credit: Swen Follak (all) | | |
| **Question S7** | *Asclepias syriaca* | |
| **Additional Information** | Pictures of species to aid recognition. | |
| 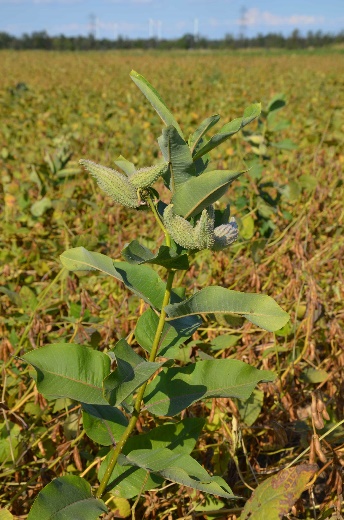 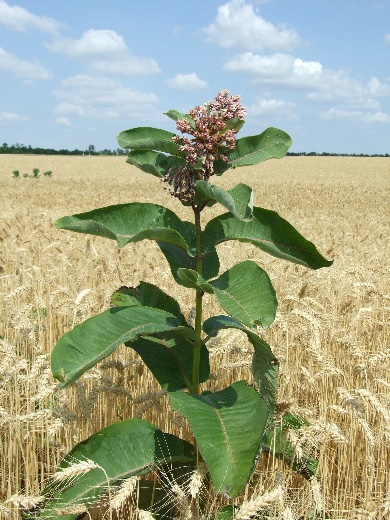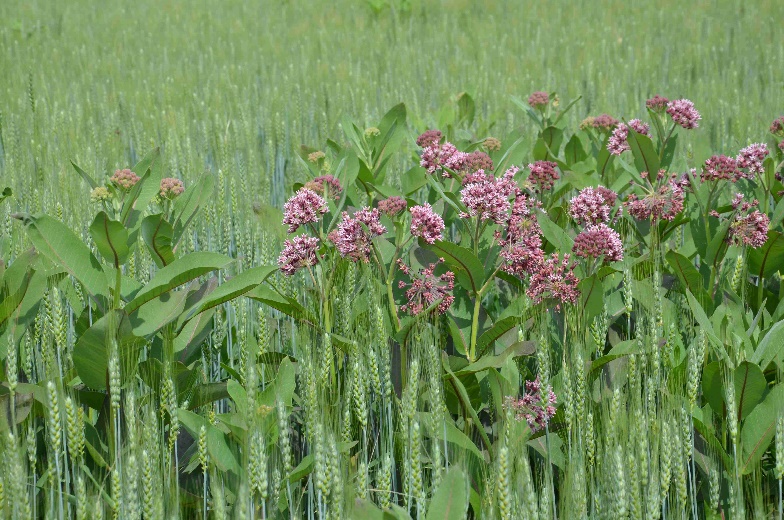  image credit: Swen Follak (all) | | |
| **Question S8** | Descurainia sophia | |
| **Additional Information** | Pictures of species to aid recognition. | |
| 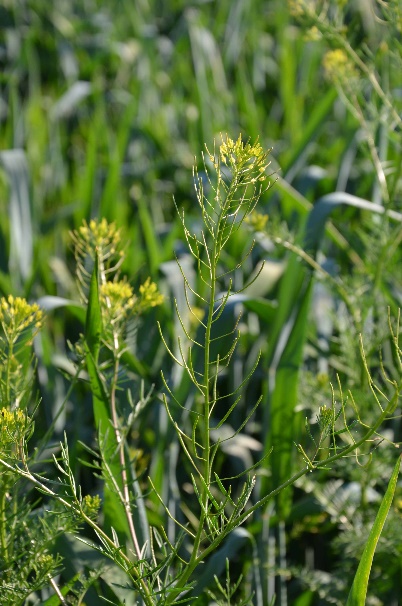 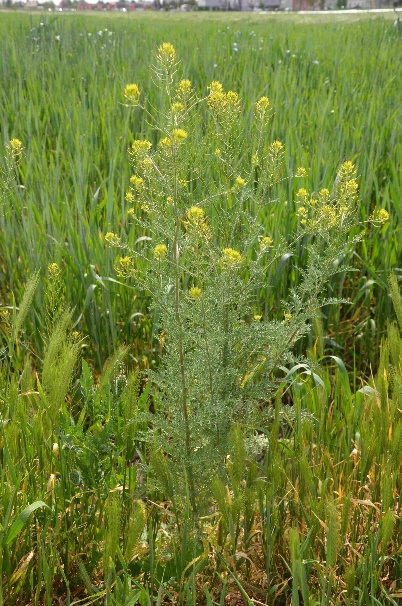 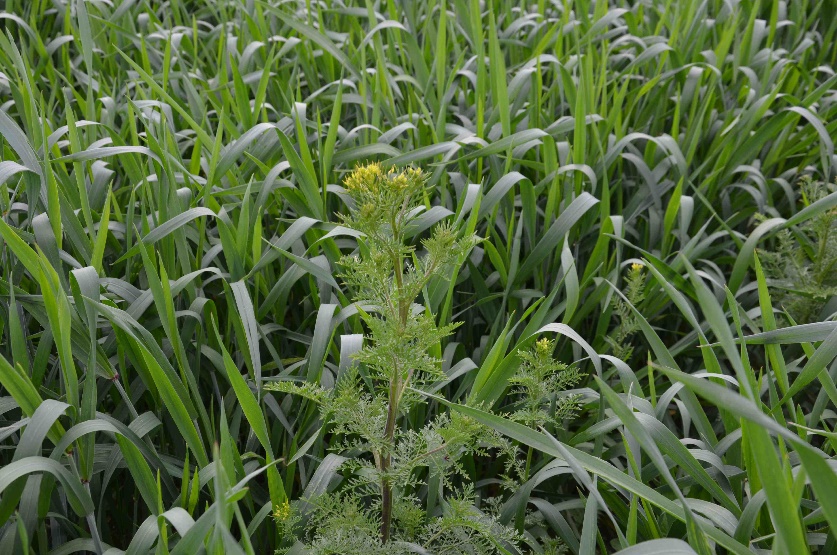  image credit: Swen Follak (all) | | |
| **Question S9** | Abutilon theophrasti | |
| **Additional Information** | Pictures of species to aid recognition. | |
| 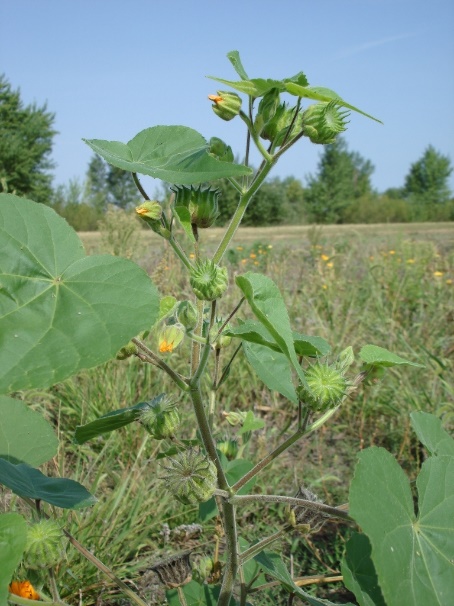 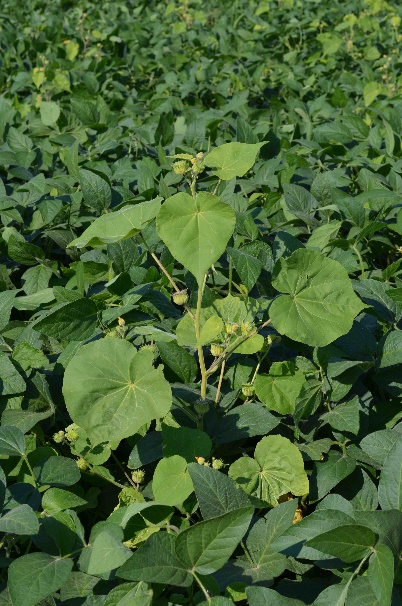 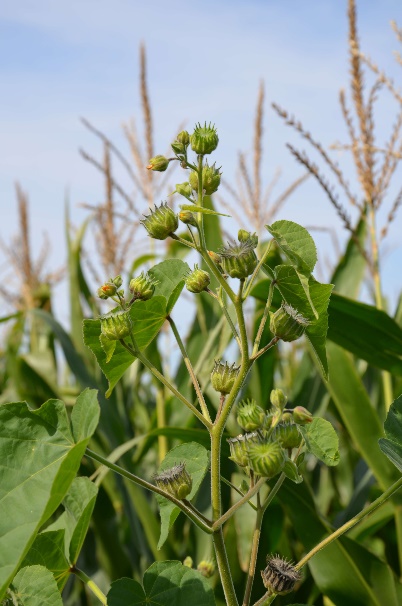  image credit: Swen Follak (all) | | |
| **Question S10** | *Fallopia* spp. | |
| **Additional Information** | Pictures of species to aid recognition. | |
| 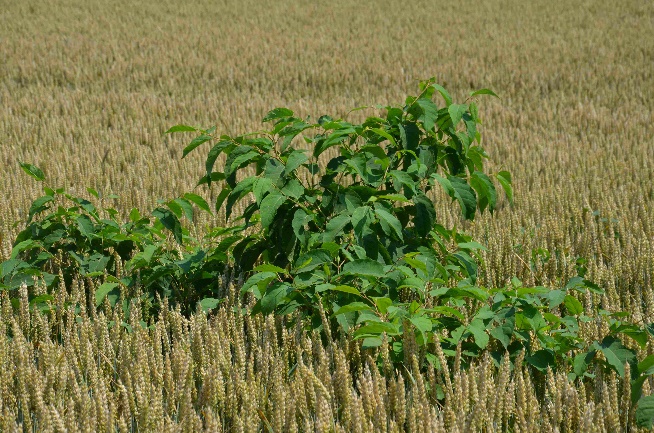 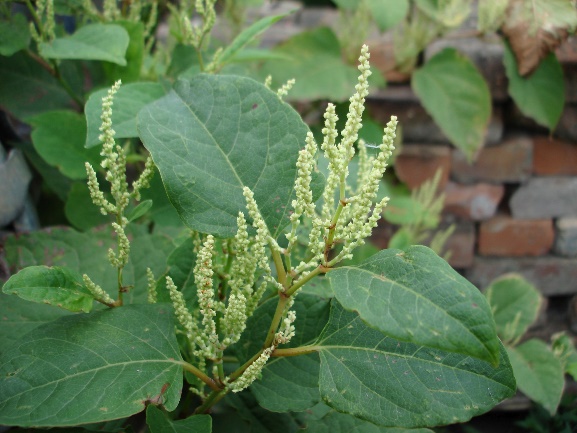  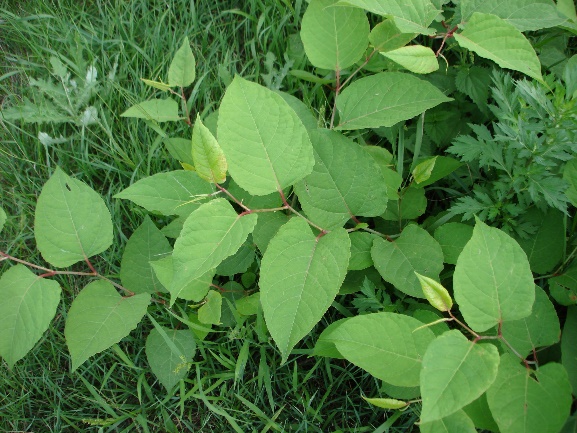  image credit: Swen Follak (all) | | |
| **Question S11** | *Panicum* spp. | |
| **Additional Information** | Pictures of species to aid recognition. | |
| 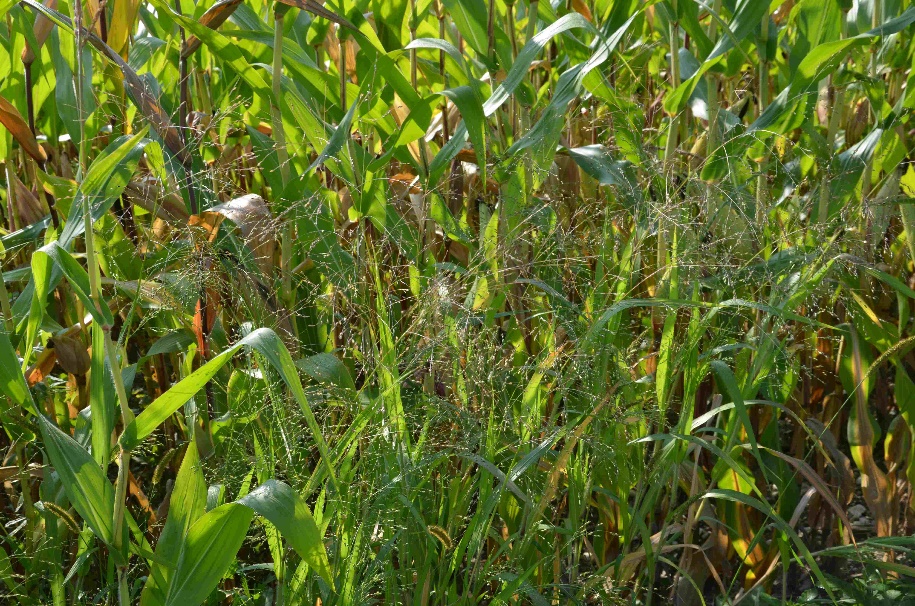 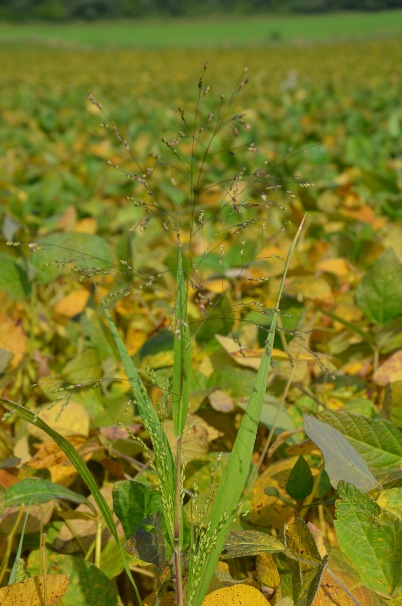  image credit: Swen Follak (all) | | |
| **Question S12** | *Amaranthus* spp. | |
| **Additional Information** | Pictures of species to aid recognition. | |
| 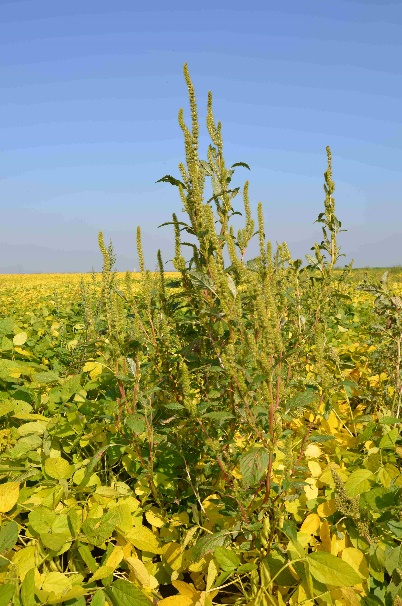 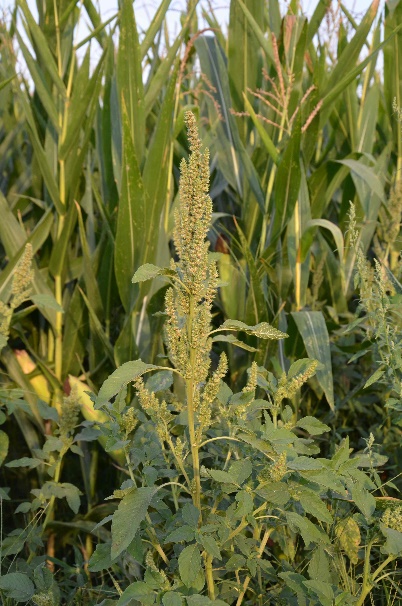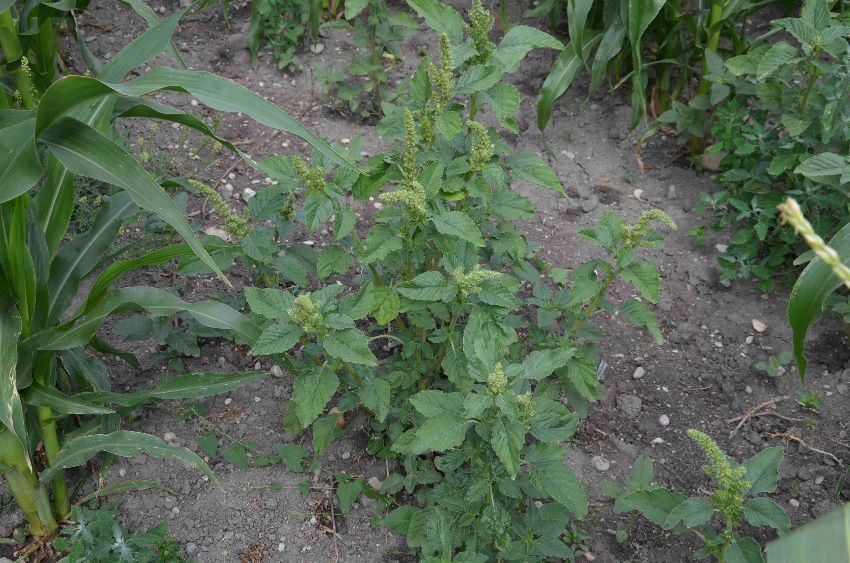  image credit: Swen Follak (all) | | |
| **Question S13** | *Solanum carolinense* | |
| **Additional Information** | Pictures of species to aid recognition. | |
| 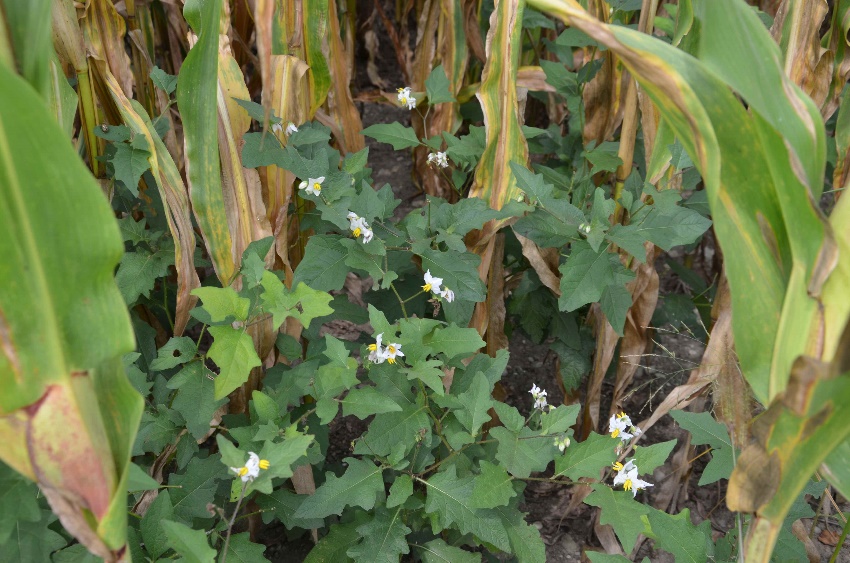 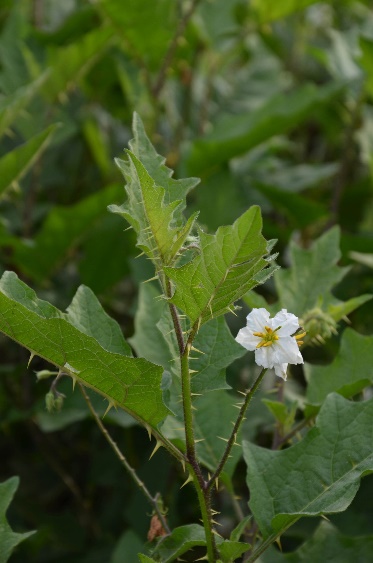 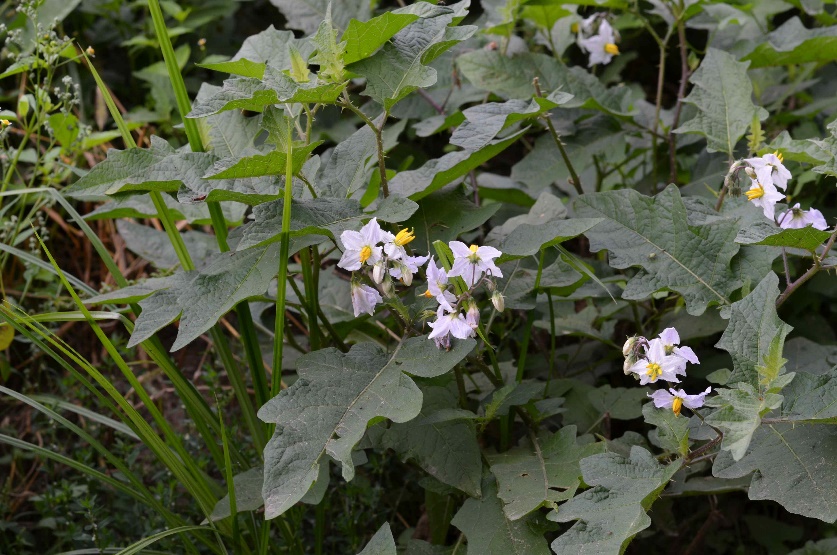  image credit: Swen Follak (all) | | |
| **Question S14** | *Helianthus tuberosus* | |
| **Additional Information** | Pictures of species to aid recognition. | |
| 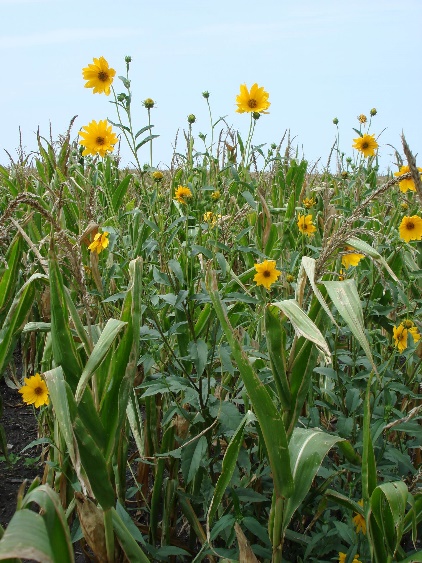 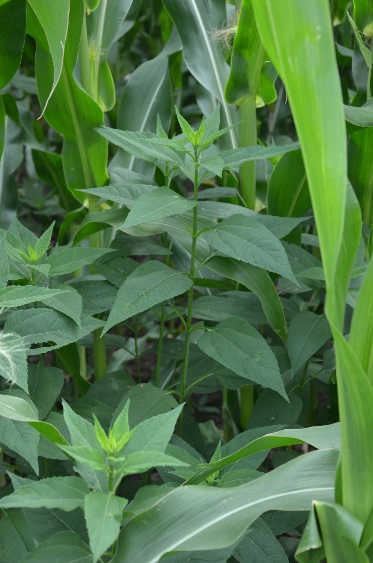 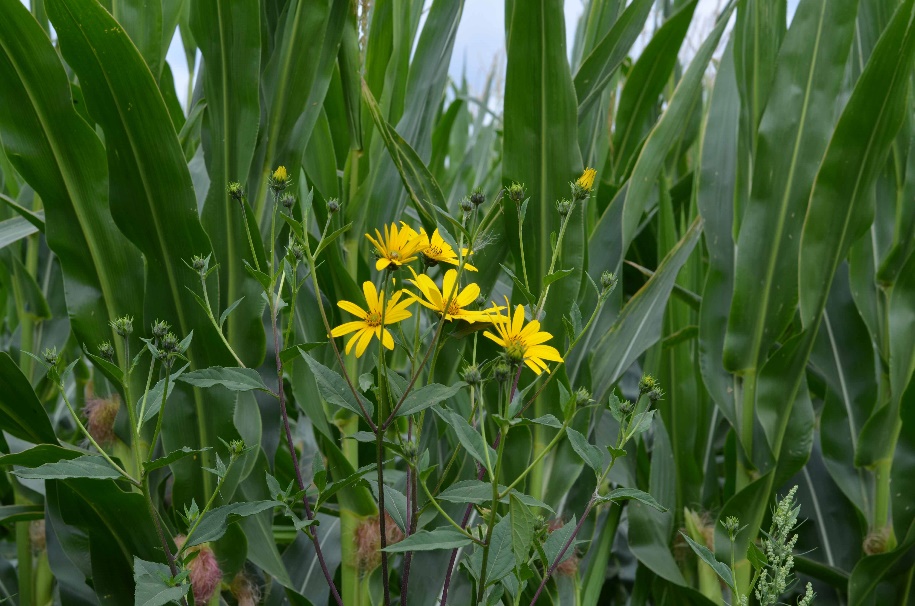  image credit: Swen Follak (all) | | |
| **Question S15** | Lactuca serriola | |
| **Additional Information** | Pictures of species to aid recognition. | |
| 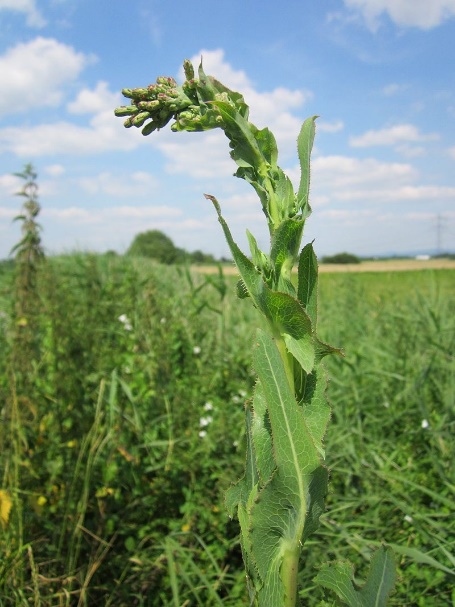 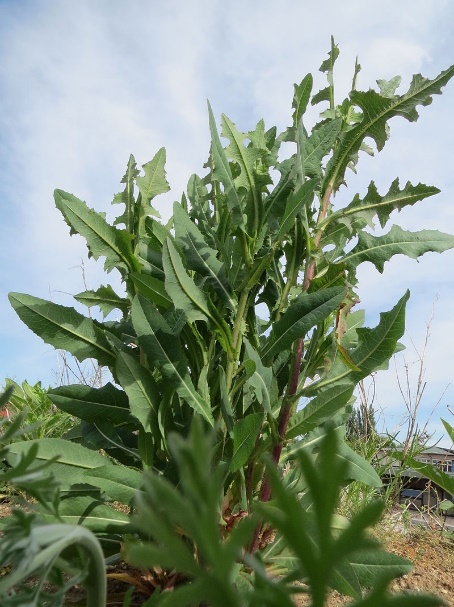 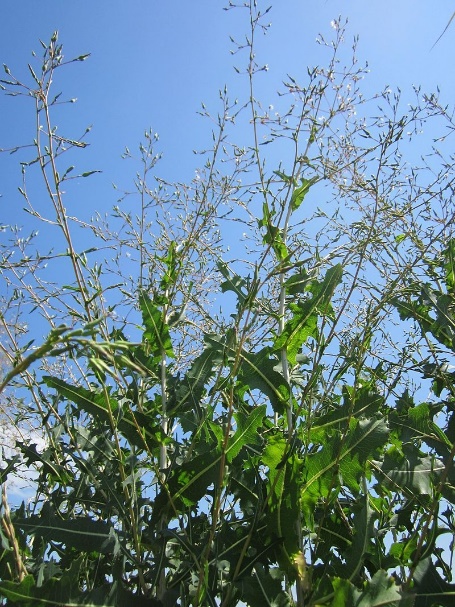  image credit: AnRo via wikicommons (all) | | |
| **Question M1-M15**  Questioins aimed at the estimated management effort of selected emerging weeds (Table 2) on resondents' farms. | **German (original)** | **English (translated)** |
|  | Wie schätzen Sie den Aufwand für die Bekämpfung der ausgewählten Unkräuter ein? | How large do you estimate the management effort for this species? |
| **Answer type** | Scale from "little to no management effort" to "high management effort" | |
| **Additional Information** | All questions come from filter questions for questions S1-S15 regarding the management estimate for emerging weed species (i.e., only farmers that had these species on their land were allowed to estimate management effort). The same pictures were as for questions S1-S15 were used to remind respondents of the species. | |

| **Question M1** | *Ambrosia artemisiifolia* | |
| --- | --- | --- |
| **Additional Information** | Pictures of species to aid recognition. | |
| **Question M2** | *Cyperus esculentus* | |
| **Additional Information** | Pictures of species to aid recognition. | |
| **Question M3** | *Xanthium strumarium* | |
| **Additional Information** | Pictures of species to aid recognition. | |
| **Question M4** | *Datura stramonium* | |
| **Additional Information** | Pictures of species to aid recognition. | |
| **Question M5** | *Phytolacca americana* | |
| **Additional Information** | Pictures of species to aid recognition. | |
| **Question M6** | *Sorghum halepense* | |
| **Additional Information** | Pictures of species to aid recognition. | |
| **Question M7** | *Asclepias syriaca* | |
| **Additional Information** | Pictures of species to aid recognition. | |
| **Question M8** | *Descurainia sophia* | |
| **Additional Information** | Pictures of species to aid recognition. | |
| **Question M9** | *Abutilon theophrasti* | |
| **Additional Information** | Pictures of species to aid recognition. | |
| **Question M10** | *Fallopia* spp. | |
| **Additional Information** | Pictures of species to aid recognition. | |
| **Question M11** | *Panicum* spp. | |
| **Additional Information** | Pictures of species to aid recognition. | |
| **Question M12** | *Amaranthus* spp. | |
| **Additional Information** | Pictures of species to aid recognition. | |
| **Question M13** | *Solanum carolinense* | |
| **Additional Information** | Pictures of species to aid recognition. | |
| **Question M14** | *Helianthus tuberosus* | |
| **Additional Information** | Pictures of species to aid recognition. | |
| **Question M15** | *Lactuca serriola* | |
| **Additional Information** | Pictures of species to aid recognition. | |
| **Question 4**  Question aimed at utilized management efforts. | **German (original)** | **English (translated)** |
|  | Auf meinem Betrieb bekämpfe bzw. unterdrücke ich Unkraut durch... | On my farm I use the following methods to manage weeds... |
| **Answer type** | multiple choice including 3 open text fields, "other 1-3") | |
| **Answer** | **German (original)** | **English (translated)** |
|  | - Herbizide - mechanische Unkrautbekämpfung (z.B. Striegel, Hacke) - manuelle Entfernung - Fruchtfolge - Einsatz von zertifiziertem Saatgut - Auswahl konkurrenzstarker Sorten - Saatdichte - Reihenweite - Zwischenfrüchte - wendende Grundbodenbearbeitung - Stoppelbearbeitung - thermische Unkrautbekämpfung - andere Maßnahmen 1-3 | - herbicides - mechanical control (e.g., hoe) - manual removal - crop rotation - use of certified seed - choice of competitive cultivars - seed density - row width - intercrops - tillage - stubble treatment - thermal control - other 1-3 |
| **Question 5**  Question aimed at the estimated financial weed management effort in major land use types. | **German (original)** | **English (translated)** |
|  | Ihrer Einschätzung nach, wie hoch ist der durchschnittliche finanzielle Aufwand für die Unkrautbekämpfung auf Ihrem Betrieb pro Hektar? | Can you estimate the financial effort (in €/ha) of weed management on your farm for the following crop types? |
| **Answer type** | entry of min and max estimate (*min* and *max* below)   - fields from *min* €/ha to *max* €/ha - orchards from *min* €/ha to *max* €/ha - vineyards from *min* €/ha to *max* €/ha | |

| **Question 6**  Statements aimed at farmers perceptions of changes in the weed flora and the reasons behind them. | **German (original)** | **English (translated)** |
| --- | --- | --- |
|  | Als Landwirt*in haben Sie die größte Erfahrung hinsichtlich der Unkräuter auf Ihrem Betrieb. Bitte bewerten Sie folgende Aussagen aus Ihrer betrieblichen Erfahrung: | As farmers, you have the most experience regarding the weeds on your land. Please rate following statements according to your experience: |
| **Answer type** | Likert scale ranging from "strongly disagree" over "neutral" to "strongly agree" | |
| **Answer** | **German (original)** | **English (translated)** |
|  | 1. Die Unkrautflora auf meinem Betrieb hat sich verändert. 2. Arten, die bisher harmlos waren, treten jetzt vermehr schädlich auf. 3. Es sind neue Unkräuter, die noch nie auf meinem Betrieb vorkamen, aufgetreten. 4. Die Veränderung der Unkrautflora hängt mit dem Klimawandel zusammen. 5. Die Veränderung der Unkrautflora hängt mit der Bewirtschaftungsweise zusammen. 6. Über das Auftreten neuer Unkräuter bin ich ausreichend informiert. 7. Auf meinem Betrieb habe ich mit Unkräutern zu tun, die ich mit den mir bekannten Methoden kaum kontrollieren kann. 8. Extremes Wetter (Dürre, Überschwemmung, Hagel usw.) erschweren mir die Unkrautbekämpfung. 9. Die Veränderung der Unkrautflora hängt mit dem überbetrieblichen Maschineneinsatz zusammen. | 1. The weed flora on my land has changed. 2. Once harmless weeds now cause damage. 3. Weeds that have never been on my land are now present. 4. The changes in the weed flora are due to climate change. 5. The changes in the weed flora are due to management practices. 6. I feel adequately informed about new and emerging weeds. 7. I have weeds on my land, I can barely control with the usual methods. 8. Extreme weather (e.g., storms, drought) impede weed management. 9. The changes in the weed flora are due to sharing machines between farms. |
| **Question 7**  Questions regarding respondents' perspective of weeds in the context of biodiversity. | **German (original)** | **English (translated)** |
|  | Bitte geben Sie Ihre Meinung zu folgenden Aussagen an. |  |
| **Answer type** | Likert scale ranging from "strongly disagree" over "neutral" to "strongly agree" | |
| **Answer** | **German (original)** | **English (translated)** |
|  | 1. Eine geringe Verunkrautung zugunsten der Biodiversität kann ich hinnehmen. 2. Ich finde, man muss harmlose Unkräuter ("Beikräuter") klar von schädlichen Unkräutern unterscheiden. 3. Manche Unkräuter haben auch positive Auswirkungen. 4. Biodiversitätsflächen (z.B. Blühstreifen, unbehandelte Ackerrandstreifen) leisten einen wichtigen Beitrag zur Biodiversität | 1. I can tolerate some weeds for the sake of biodiversity. 2. I think noxious weeds should be clearly separated from harmless weeds. 3. Some weeds have positive effects. 4. Biodiversity-rich sites within agricultural areas are important for biodiversity. |
| **Questions D1 – D5**  Questions regarding demography and farm location to further characterize respondents. | **Answers** | |
| Age | (years) | |
| Where is your farm? | (post code) | |
| Job experience | (years) | |
| How large is your farm? | (in hectare) | |
| What is the highest level of education you've acquired? | **German** | **English** |
|  | - Ausbildung am Betrieb - Landwirtschaftliche Fachschule - Landwirtschaftlich*r Meister*in - Landwirtschaftliches Studium - Andere - keine Angabe | - on-farm education - agricultural school - agricultural "master" - agricultural university degree - other (free text) - prefer not to say |

Table S 2: Stakeholders that helped in distributing the *survey* invitation and reach estimates (bold... known reach of stakeholder (e.g. via newsletter subscriptions etc.); non-bold.... estimated reach by stakeholder)

| Name/Organization | Description | Reach |
| --- | --- | --- |
| Adamah | organic/regional food cooperative marketing | **250** |
| AMA | Austrian agricultural quality control organization | **10000** |
| Arche Noah | organic seed retailer | 100 |
| BIO AUSTRIA | Austrian organic farming organization | **4700** |
| CVL-info | mailing list of first authors' institution | 50 |
| CVL-facebook | Facebook page of first authors' institution | 50 |
| demeter | organic certification organization | 500 |
| Donau Soja | Austrian soybean stakeholder organization | **50** |
| IG Erdäpfelbau | Austrian potato farming stakeholder organization | **400** |
| LGV | Austrian vegetable farming cooperative | **160** |
| LWK Bgld | Chamber of Agriculture Burgenland | 1000 |
| LWK NÖ | Chamber of Agriculture Lower Austria | **2000** |
| LWK OÖ | Chamber of Agriculture Upper Austria | **3000** |
| LWK Sbg | Chamber of Agriculture Salzburg | 500 |
| LWK Stmk | Chamber of Agriculture Styria | **3000** |
| LWK T | Chamber of Agriculture Tyrol | 500 |
| LWK Vbg | Chamber of Agriculture Vorarlberg | 500 |
| Markta | organic/regional food cooperative marketing | 100 |
| ÖAIP | Austrian Association of Integrated Plant Protection | 100 |
| Pflanzenschutzbeauftragte | Austrian regional plant protection experts | 100 |
| Rübenbauernverein | Austrian beet farmer stakeholder organization | 100 |
| Suske Consulting | consulting firm with ties to farming and conservation | 50 |
| Weinbauernverbände | Austrian vintner association | 500 |
|  |  | 27710 |
